# Stunting is associated with blood lead concentration among Bangladeshi children aged 2-3 years

**DOI:** 10.1186/s12940-016-0190-4

**Published:** 2016-11-04

**Authors:** Kelsey M. Gleason, Linda Valeri, A. H. Shankar, Md Omar Sharif Ibne Hasan, Quazi Quamruzzaman, Ema G. Rodrigues, David C. Christiani, Robert O. Wright, David C. Bellinger, Maitreyi Mazumdar

**Affiliations:** 1 Department of Environmental Health - EOME Program, Harvard T.H. Chan School of Public Health, Landmark Center, 401 Park Drive, 3rd Floor East, Boston, MA 02215 USA; 2 Department of Psychiatry (Biostatistics), McClean Hospital, Belmont Campus, North Belknap, Room 310A, Belmont, MA 02478 USA; 3 Department of Psychiatry, Harvard Medical School, Boston, MA USA; 4 Department of Nutrition, Harvard T.H. Chan School of Public Health, 655 Huntington Avenue, Building 2, Room 331A, Boston, MA 02115 USA; 5 Dhaka Community Hospital, 190/1, Wireless Railgate Bara Moghbazar, Dhaka, 1217 Bangladesh; 6 Department of Neurology, Boston Children’s Hospital, 651 Huntington Avenue, FXB, Room 102, Boston, MA 02115 USA; 7 Department of Preventive Medicine, Mount Sinai School of Medicine, 17 East 102nd Street, New York, NY 10029 USA; 8 Department of Neurology, Harvard Medical School, 300 Longwood Ave, Children’s Hospital Farley Basement Box 127, Boston, MA 02115 USA

**Keywords:** Bangladesh, Children, Environmental toxins, Heavy metals, Lead exposure, Neurodevelopment, Stunting

## Abstract

**Background:**

Lead toxicity is of particular public health concern given its near ubiquitous distribution in nature and established neurotoxicant properties. Similar in its ubiquity and ability to inhibit neurodevelopment, early childhood stunting affects an estimated 34 % of children under 5 in low- and middle-income countries. Both lead and stunting have been shown to be associated with decreased neurodevelopment, although the relationship between these childhood burdens is underexplored. The association between lead exposure and stunting has been previously established, yet limited data are available on susceptibility windows.

**Methods:**

Whole blood lead samples were collected from rural Bangladeshi children at delivery (umbilical cord blood) and at age 20–40 months (fingerstick blood). Stunting was determined using the Child Growth Standards developed from the World Health Organization Multicentre Growth Reference Study. Children with height for age < -2 z-scores below the median of the WHO Child Growth Standards were classified as stunted in all analyses.

**Results:**

Median (IQR) umbilical cord and fingerstick blood lead levels were 3.1 (1.6–6.3) μg/dl and 4.2 (1.7–7.6) μg/dl, respectively. In adjusted multivariable regression models, the odds of stunting at 20–40 months increased by 1.12 per μg/dl increase in blood lead level (OR = 1.12, 95 % CI: 1.02–1.22). No association was found between cord blood lead level and risk of stunting (OR = 0.97, 95 % CI: 0.94–1.00).

**Conclusions:**

There is a significant association between stunting and concurrent lead exposure at age 20–40 months. This association is slightly attenuated after controlling for study clinic site. Additional research including more precise timing of lead exposure during these critical 20–40 months is needed.

**Electronic supplementary material:**

The online version of this article (doi:10.1186/s12940-016-0190-4) contains supplementary material, which is available to authorized users.

## Background

Children are particularly susceptible to, and increasingly confronted by, a myriad of health burdens resulting from exposure to environmental toxins. Toxic metals such as lead (Pb) are of particular public health concern given their near ubiquitous distribution and persistency in nature [1]. Although lead naturally occurs in the earth’s crust, its presence in the human environment has increased steadily with the growth of industry [2].

Having been found in foodstuffs [3–7] and children’s toys [8], lead has previously been described as a “multimedia pollutant” due to its multitude of historical and current uses and pathways of exposure [9]. It is a well-documented environmental neurotoxicant whose adverse effects have been highlighted by recent literature citing no threshold below which lead has no effect on the developing brain [10–12].

The inconsistent regulations surrounding the use of lead across the globe have resulted in disproportionate exposure potentials, with developing countries at the highest risk for heavy metal over-exposure [13]. Further compounding this disparity in global exposure is the overwhelming prevalence of chronic stunting, which affects an estimated 34 % of children younger than 5 years in low- and middle-income countries [14]. Growth potential has been shown to be similar across all countries, indicating that stunting occurs as a result of chronic malnutrition rather than genetic differences [15, 16]. More than 200 million children under 5 years of age are currently estimated to fail to reach their cognitive development potential because of poor health, poverty, and poor nutrition [17]. In addition, similar to the neurodevelopmental effects of lead, a growing body of evidence has shown that stunting, an indicator of chronic malnutrition, is associated with reduced educational attainment [17] and neurodevelopment including lower achievement levels, IQ, and higher order cognitive processing [18–20].

Bangladesh is one such developing country that is particularly vulnerable to high levels of lead exposure due to the country’s poorly enforced environmental regulations [21, 22] and high prevalence of malnutrition [23]. The specific objectives of this study were to investigate the association between blood lead level and chronic malnutrition among children in rural Bangladesh and to identify a critical window of exposure for the effect of lead on chronic malnutrition. We hypothesized that concurrent rather than *in utero* lead exposure would be more highly associated with chronic malnutrition among children in rural Bangladesh.

## Methods

### Subject selection

Details of the recruitment strategy, eligibility criteria, and sample collection from children participating in this study have been published previously [24, 25]. Briefly, participants were members of a longitudinal birth cohort established in rural Bangladesh to study the health effects of prenatal and early childhood exposures to metals in the environment. Between 2008 and 2011, pregnant women in the Sirajdikhan and Pabna Upazilas of Bangladesh were enrolled and followed through pregnancy. Gestational age was determined by first trimester ultrasound. Between 2010 and 2013, when children were aged 12 to 40 months, healthcare workers from Dhaka Community Hospital (DCH) invited families to enroll their children in follow-up studies; all children born to mothers in the original study were eligible. Informed consent was provided by the parents of all participants before enrollment. The Committee for Clinical Investigation at Boston Children’s Hospital (BCH) ceded review of this study to the Harvard T.H. Chan School of Public Health (Harvard Chan School). The Human Research Committees at the Harvard Chan School and DCH approved this study.

A total of 1613 pregnant women were enrolled in the reproductive outcomes study. Over the course of the study, 123 women were lost to follow-up, 125 women no longer wished to participate in the study, and 132 pregnancies resulted in miscarriage, 75 in stillbirth, and 5 in twin births, resulting in 1153 mothers and their children participating in this study. A total of 815 children participated in complete neurodevelopmental assessments; 624 of these included fingerstick blood lead measurements at age 20 to 40 months. Complete data for cord blood lead concentration, fingerstick blood lead concentration, water metal concentrations, and stunting outcomes were available for 618 children, and this comprises the study sample for the current analysis.

### Environmental exposure assessment

Lead exposure was assessed using blood samples collected at two time points: umbilical cord blood at birth, representing prenatal lead exposure, and fingerstick blood at 20 to 40 months of age, representing early childhood exposure. Umbilical cord blood samples were collected in trace metal-free tubes and were analyzed for lead at the Trace Metals Laboratory at the Harvard Chan School. Umbilical cord blood samples were first weighed (~1 g) and digested for 24 h in 2 mL of concentrated nitric acid. The samples were then treated with 1 mL of 30 % hydrogen peroxide per 1 g of blood and left overnight. Samples were subsequently diluted to 10 mL with deionized water. Blood lead concentrations were measured using a dynamic reaction cell-inductively coupled plasma mass spectrometer (DRC-ICP-MS, DRC II, Perkin Elmer). Analyses were performed using an external calibration method, with lutetium as the internal standard for lead. Continual calibration standards and a standard reference solution (NIST 1643e: Metals in Water) were used to monitor precision and accuracy of the analysis. The final reported value in this study was an average of 5 replicate measurements for each individual sample.

Fingerstick blood lead concentrations were measured using portable LeadCare II instruments (Magellan Diagnostics, Billerica, MA, USA) that have a reportable range of 3.3–65 μg/dL. A subset of samples was obtained via venipuncture for confirmation. Before testing, study staff washed the children’s hands to avoid contaminating the blood sample with lead from the skin. Each child’s finger was pricked with a single-use lancet, and a 50 μL capillary tube was filled after discarding the first droplet. Daily quality-assurance measures were performed according to the user’s manual.

Quality control measures for blood measurements included analysis, procedural blanks, duplicate samples, spiked samples, and analysis of a certified reference material (NIST 955b: bovine blood for lead) to monitor for contamination, accuracy, and recovery rates. Recovery rates for lead in quality control and spiked samples ranged from 81 to 108 %, and precision was measured as percent relative standard deviation (SD), with a result of less than 3 % for lead. The average limit of detection (LOD) was 0.2 μg/dL.

Water samples were collected at four time points: 1) enrollment of mothers, 2) 1 month postpartum, 3) 12 months of age, and 4) 20 to 40 months of age. The average arsenic/manganese exposure value across these four visits was used in all analyses [26]. Arsenic and manganese concentrations were measured using water samples collected from tube wells identified by enrolled mothers as the household’s primary source of drinking water. Water samples were collected in 50 ml polypropylene tubes (BD Falcon, BD Bioscience, Bedford, MA), preserved with Reagent Grade nitric acid (Merk, Germany) to a pH < 2, and stored at room temperature. Analysis of all arsenic and manganese samples was conducted using inductively coupled plasma mass-spectrometry (ICP-MS) (Spectrum Analytical, Inc., Agawam, MA USA) following U.S. Environmental Protection Agency (EPA) method 200.8 [27].

### Stunting

Stunting status of children was determined using the World Health Organization (WHO) macros (Version 3.2.2). This software utilizes the Child Growth Standards developed from the WHO Multicentre Growth Reference Study (MGRS) [16]. These standards aim to provide a single international standard of physiological growth for all children from birth to 5 years of age. Applying this international standard, children with height for age < -2 z-scores below the median of the WHO Child Growth Standards were classified as stunted in all analyses.

### Covariates

At the time of enrollment of the mothers, trained study staff administered a questionnaire targeting maternal demographics and medical history. Data on education, smoking history, and maternal weight were also collected during this visit.

Infant sex and length were recorded at birth. A food frequency questionnaire was administered prior to the birth of the child. A measure of maternal protein intake during pregnancy was derived from frequency of consumption (times consumed per week) of fish, meat, and egg, to account for potential confounding by mother’s nutritional status. Protein intake was coded as a categorical variable indicating weekly protein consumption as high (>26 units of protein), medium (12.5–26 units of protein), and low (<12.5 units of protein).

During the follow-up study, trained interviewers administered the Home Observation for Measurement of the Environment (HOME) Inventory that had been previously translated and adapted for use in Bangladesh [28]. The HOME Inventory measures the quality and quantity of stimulation and support available to the child in the home environment. The HOME Inventory is comprised of 7 sub scales that were summed into one composite HOME Inventory variable.

At the 20- to 40-month home visit, trained study staff also administered the Edinburgh Postnatal Depression Scale (EPDS) to mothers [29] as a measure of depressive symptoms.

### Statistical methods

All statistical procedures were performed using RStudio version 0.98.1087. To investigate the potential for differences in demographics and exposure profiles between the two study sites, descriptive statistics and metal distributions were examined for all variables by clinic. We considered the presence of effect modification by study site and stratified our analysis.

A multivariate logistic regression model was constructed by including covariates selected through review of the literature. Blood lead concentration at birth (cord blood) was modeled as a continuous variable. Fingerstick blood lead concentration took a non-linear form in the association with stunting, and we modeled this variable using a quadratic effect. In this analysis, the primary outcome of interest, stunting, was modeled as a dichotomous variable, indicating the presence or absence of stunting. Maternal weight, maternal education, maternal protein intake, and HOME Inventory score were all modeled as continuous variables. Average water arsenic and manganese levels were included as continuous variables in the model to account for differences in the distribution of the metals between sites.

Initial models included EPDS as a measure of maternal depression; however, inclusion of this variable reduced our sample size by 44 % (*n* = 346), and this variable was not included in our final model. We further considered the possibility of unmeasured confounding by study site and initially included an indicator variable for clinic site in our model. After inclusion of all known predictors and confounders, including EPDS, study site was not found to be statistically significant (*p* = 0.19).

## Results

Selected demographic characteristics stratified by study site (Pabna and Sirajdikhan) are presented in Table 1. Exposure distributions of lead, arsenic, and manganese are shown in Table 2. Fingerstick blood lead concentrations obtained using the portable LeadCare II instruments were confirmed using a subset of samples obtained via venipuncture (*r* = 0.80).

**Table 1:** Demographics of population, stratified by study site

	Current Study Population (*n* = 618)	Pabna (*n* = 317, 51.3 %)	Sirajdikhan (*n* = 301, 48.7 %)
	Number (%)		
Sex			
*Male*	307 (49.7)	156 (49.2)	151 (50.2)
*Female*	311 (50.3)	161 (50.8)	150 (49.8)
Stunting Status			
*Stunted*	324 (52.4)	135 (42.6)	189 (62.2)
*Not Stunted*	294 (47.6)	182 (57.4)	112 (37.2)
Child Smoke Exposure			
*Exposed*	260 (42.1)	158 (49.8)	102 (33.9)
*Not Exposed*	358 (57.9)	159 (50.2)	199 (66.1)
Mother's education			
*Primary or less*	285 (46.1)	141 (44.5)	144 (47.8)
*Secondary or greater*	333 (53.9)	176 (55.5)	157 (52.2)
Mother's Protein			
*Low*	139 (22.5)	1 (0.3)	138 (45.8)
*Medium*	336 (54.4)	184 (58.0)	152 (50.5)
*High*	143 (23.1)	132 (41.6)	11 (3.7)
	Mean (SD)		
Birth Gestational Age (weeks)	37.9 (2.0)	37.1 (2.0)	38.8 (1.6)
*Missing*	*2*	*2*	*0*
Age (months)	28 (2.9)	27.8 (2.8)	28.3 (3.0)
Mother's Weight at Enrollment (kg)	46.6 (7.6)	45.1 (7.1)	48.1 (7.9)
Mother's Height (cm)	151.4 (5.6)	151.1 (5.1)	151.7 (6.2)
Child's Birth Weight (kg)	2.85 (0.4)	2.8 (0.5)	2.9 (0.3)
*n (%) ≤ 2.5 kg*	90 (14.6)	71 (22.4)	19 (6.3)
Child's Birth Length (cm)	46.6 (2.4)	46.9 (2.3)	46.2 (2.5)
HOME Score	42.7 (2.6)	41.5 (2.7)	44 (1.8)
*Missing*	*0*	*0*	*1*
Maternal Depression	15.2 (2.8)	15.3 (2.1)	15.1 (3.5)
*Missing*	*272*	*125*	*147*

**Table 2:** Heavy metal exposure distribution

	Total Study Population	Pabna	Sirajdikhan
	(*n* = 618)	(*n* = 317)	(*n* = 301)
	Median (IQR)		
Umbilical Cord Blood Lead (μg/dL)	3.1 (1.6–6.3)	1.6 (1.1–2.4)	6.2 (4.0–9.4)
* n (%) ≥ 5 μg/dL*	206 (33.3)	17 (5.4)	189 (62.8)
Blood Lead 20–40 months (μg/dL)	4.2 (1.7–7.6)	1.7 (1.7–3.7)	7.3 (5.3–8.2)
* n (%) ≥ 5 μg/dL*	282 (45.6)	43 (13.6)	239 (79.4)
Average Water Arsenic (μg/L)	13.4 (1.9–66.3)	34.6 (8.2–120.7)	3.3 (1.2–19.5)
Average Water Manganese (μg/L)	625.5 (368.6–1066.0)	569.0 (344.3–993.3)	750.3 (431.8–1148.0)

Examination of the stratified demographics highlights several differences between study sites. Compared to children from Pabna, children from Sirajdikhan were more likely to be stunted (62.2 % vs. 42.6 %) but were less likely to be exposed to cigarette smoke (33.9 % vs. 49.8 %). Additionally, the metal exposure profiles differ between the two study sites. Compared to children from Pabna, children from Sirajdikhan were more likely to have blood lead levels that exceed the Centers for Disease Control and Prevention (CDC) lead exposure reference level of 5 μg/dL for children aged 1–5 [30], both at birth (62.8 % vs. 5.4 %) and at 20–40 months of age (79.4 % vs. 13.6 %). Additionally, compared to children from Pabna, children from Sirajdikhan had higher average manganese exposure (median μg/L [IQR]: 750.3 [431.8–1148.0] vs. 569.0 [344.3–993.3]), but lower average arsenic exposure (median μg/L [IQR]: 3.3 [1.2–19.5] vs. 34.6 [8.2–120.7]).

Demographic characteristics of stunted versus non-stunted children were also examined (Table 3). Of all children in this study, 52.4 % were classified as stunted. As compared to their non-stunted peers, these stunted children were less likely to be exposed to cigarette smoke (38.6 % vs. 45.9 %) and less likely to have mothers who had achieved a secondary education or greater (47.5 % vs. 60.9 %). In addition, as compared to non-stunted children, stunted children had lower average water arsenic concentration (mean μg/L [sd]: 48.4 [88.0] vs. 68.1 [130.7]), but higher average water manganese exposure (mean μg/L [sd]: 813.3 [582.2] vs. 745.1 [554.6]). Stunted children also had a higher mean blood lead level at 20–40 months as compared to non-stunted children (mean μg/dL [sd]: 6.2 [5.0] 4.6 [4.1]).

**Table 3:** Demographics and metal exposures of study population, stratified by stunting status

	Total Population (*n* = 618)	
	Stunted (*n* = 324)	Not Stunted (*n* = 294)
*Demographics*	Number (%)	
Sex		
*Male*	159 (49.1)	148 (50.3)
*Female*	165 (50.9)	146 (49.7)
Stunting Status		
*Stunted*	324 (52.4)	–
*Not Stunted*	*–*	294 (47.6)
Child Smoke Exposure		
*Exposed*	125 (38.6)	135 (45.9)
*Not Exposed*	199 (61.4)	159 (54.1)
Mother's education		
*Primary or Less*	170 (52.5)	115 (39.1)
*Secondary or Greater*	154 (47.5)	179 (60.9)
Mother's Protein		
*Low*	101 (31.2)	38 (12.9)
*Medium*	168 (51.9)	168 (57.1)
*High*	55 (17.0)	88 (29.9)
	Mean (SD)	
Birth Gestational Age (weeks)	38.0 (2.0)	37.9 (2.0)
*Missing*	2	0
Age (months)	28.1 (2.9)	28.0 (2.9)
Mother's Weight at Enrollment (kg)	46.0 (7.4)	47.2 (7.8)
Mother's Height (cm)	150.4 (5.6)	152.4 (5.5)
Child's Birth Weight (kg)	2.9 (0.4)	2.9 (0.4)
Child's Birth Length (cm)	46.2 (2.7)	46.9 (2.0)
HOME Score	42.7 (2.5)	42.7 (2.7)
*Missing*	1	0
Maternal Depression	15.0 (2.9)	15.4 (2.8)
*Missing*	141	132
*Heavy Metal Exposures*	Mean (SD)	
Umbilical Cord Blood Lead (μg/dL)	5.1 (5.2)	4.9 (7.8)
Blood Lead 20–40 months (μg/dL)	6.2 (5.0)	4.6 (4.1)
Average Water Arsenic (μg/L)	48.7 (88.0)	68.1 (130.7)
Average Water Manganese (μg/L)	813.3 (582.1)	745.1 (554.6)

Because the sample under study is a subset of a larger population-based cohort, we evaluated selection bias by comparing the distribution of participant demographics and exposure profiles between our included study population and the excluded population. Evaluation of the distribution of demographics between our study population and the excluded population show important differences only between the age of the child at the second lead collection time point (*p* = 0.03) and the measure of mother’s protein consumption (*p* < 0.01) (see Additional file 1: Table S1). No important differences were found in exposure distributions for blood lead at either time point, for water arsenic, or for water manganese (see Additional file 1: Table S2).

Table 4 displays the results of multivariable regression models for the association between lead exposure at birth and at 20 to 40 months of age and stunting at 20 to 40 months. In both models, lead exposure at 20 to 40 months was modeled allowing for a quadratic association. Of the confounders considered, this model revealed the strongest predictors of stunting status to be mother’s education, height, and protein intake during pregnancy. No other potential confounders included in the model were found to be significant predictors of stunting status.

**Table 4:** Associations between blood lead level and stunting status

	*Unadjusted for Clinic Site*		*Adjusted for Clinic Site and Maternal Depression*	
	*OR*	*CI*	*OR*	*CI*
Blood Lead Level				
*Umbilical Cord Blood*	0.98	0.95–1.01	0.97	0.93–1.00
*20–40 months*	1.12	1.02–1.22	1.15	1.00–1.33
Mother's Protein^a^	0.60	0.44–0.80	0.7	0.42–1.14
Mother's Education	0.78	0.63–0.96	0.71	0.53–0.94
Mother's Weight^b^	0.99	0.96–1.01	0.99	0.95–1.02
HOME Score^b^	0.96	0.89–1.03	0.97	0.88–1.06
Maternal Depression^b^	–	–	0.97	0.89–1.06
Smoke Exposure	0.82	0.58–1.17	0.94	0.59–1.51
Mother's Height	0.94	0.91–0.97	0.93	0.88–0.97
Average Water Manganese	1.00	1.00–1.00	1.0	1.00–1.00
Average Water Arsenic	1.00	1.00–1.00	1.0	1.00–1.00
Clinic Site				
Sirajdikhan	–	–	*reference*	
Pabna	–	–	0.57	0.27–1.18

^a^A measure of mother's protein was derived from frequency (times consumed per week) of fish, meat and egg consumption

^b^These measurements were taken at enrollment of the mothers, which occurred at approximately 18 weeks into their pregnancy

**Fig. 1:**
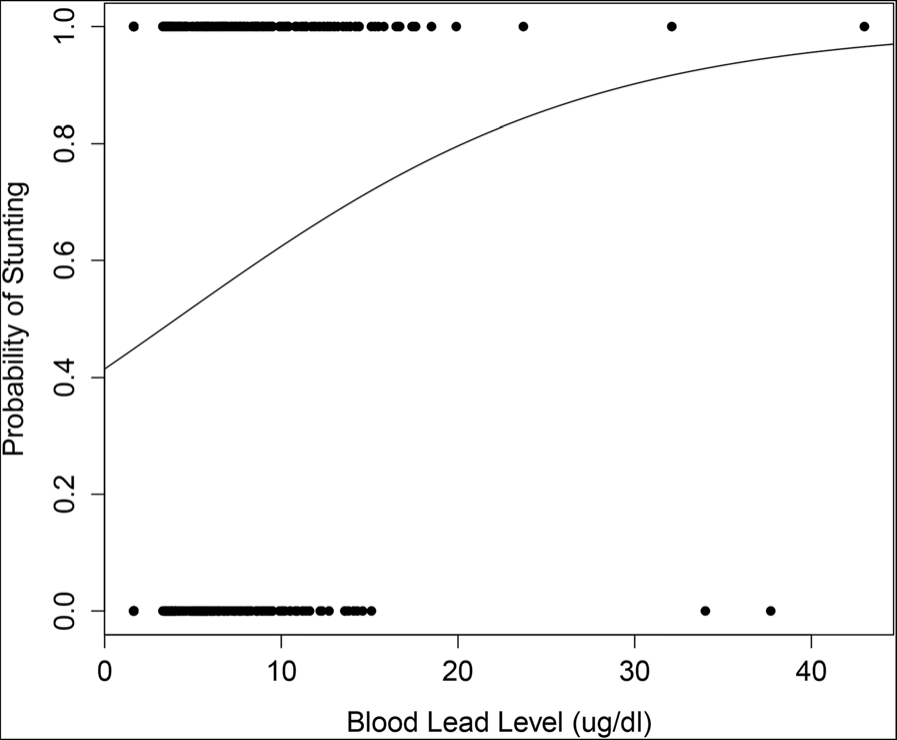
Predicted probability of stunting due to lead exposure at 20-40 months

The association between concurrent lead exposure and stunting was found to be non-linear, with an increasing probability of stunting at higher lead levels (Fig. 1). These results further indicate a significant positive association between blood lead at 20 to 40 months and stunting at 20 to 40 months (OR = 1.12; 95 % confidence interval [CI]: 1.02–1.22). No significant association was found between blood lead level at birth and stunting at 20 to 40 months (OR = 0.98; 95 % CI: 0.95–1.01). The odds of stunting at 20 to 40 months increase by 1.12 per one unit increase from zero in blood lead level at 20 to 40 months.

For interpretability, an additional logistic regression model was used to assess the relationship between an increase from the first and third quartile of blood lead at 20 to 40 months and stunting. Results of this model indicate that those children who have a blood lead level greater than the third quartile of exposure at 20 to 40 months (7.6 μg/dL) have 2.6 (95 % CI: 1.4–5.0) greater odds of being stunted as compared to children who have a blood lead level below the first quartile of exposure at 20 to 40 months (1.7 μg/dL).

A sensitivity analysis was conducted by adding an indicator variable for clinic site to the previously described multivariable logistic regression model. Results from this clinic-adjusted model again indicate a non-linear relationship between blood lead at 20 to 40 months and stunting status. These results support the findings from the clinic-unadjusted model, again indicating a positive association between blood lead at 20 to 40 months and stunting at 20 to 40 months (OR = 1.15). This result, however, was found to be borderline statistically significant (*p* = 0.06; 95 % CI: 1.0–1.33). Again, no statistically significant association was found between blood lead level at birth and stunting at 20 to 40 months (OR = 0.97; 95 % CI: 0.93–1.00). Adjusting for clinic, the odds of stunting at 20 to 40 months increase by 1.15 per one unit increase in blood lead level at 20 to 40 months.

## Discussion

Results of this study demonstrate a positive association between concurrent blood lead concentration and stunting at age 20 to 40 months. *In utero* lead exposure was also associated with stunting at age 20 to 40 months; however, this association was not as strong and was not statistically significant. This suggests that early childhood may be a critical window for the effect of lead on stunting. Albeit slightly attenuated, this association was shown to persist even after controlling for study clinic site, providing further evidence for the robustness of this finding.

The positive association between early life lead exposure and stunting has been reported previously [31–34], but several studies have cast doubt upon this relationship by reporting no association between early life lead exposure and stunting or inhibited vertical growth [35]. Attempts at investigating the relationship between multiple insults of lead exposure during early life and stunting have also reported inconsistent findings, with several studies reporting insignificant findings [33], including three longitudinal studies [36–38]. Other longitudinal studies, however, have reported significant negative effects of lead on the overall growth of children. One such study, the Mexico City Prospective Lead Study, highlights the negative association between childhood head circumference at 6 months and prenatal maternal blood lead levels [39]. Another study reported an association between children’s lead levels and reduction in stature at age 15 months, yet only among those children whose mothers had a prenatal lead level greater than 7.7 μg/dL [40].

Failure to achieve full growth potential, particularly in the form of stunting, has been shown to compromise overall childhood development, resulting in both immediate and long-term negative health outcomes. Because it reflects the accumulated, permanent, and long-term effects of deficiency in early childhood nutrition, stunting is widely considered the best epidemiological indicator for assessing chronic malnutrition [41]. Early life stunting has been shown to be associated with increased insulin resistance [42], altered fat metabolism [43], increased risk for chronic disease [44], impaired immune function [45], and increased risk of hypertension and cardiovascular disease [46]. Compounding these later life morbidities is the strong body of evidence consistently demonstrating the association between stunting and cognitive deficits [18, 20, 47]. In the absence of intervention, this cognitive delay resulting from childhood stunting has been found to persist in later life [17]. Research from randomized trials, however, shows that improving child nutritional status and growth, is associated with concurrent benefits in motor development, mental development, and overall cognitive ability [48–50], yet children who experience early life stunting fail to achieve the same cognitive development as their never-stunted peers [49, 51].

Although the positive association between early life lead exposure and stunting has been established in the literature, little research has been done to understand potential windows of exposure for the susceptibility of stunting due to lead. A large body of literature exists to identify periods of opportunity for interventions to mitigate the effects of early life stunting [52, 53], yet such literature excludes the effect of lead on stunting. By identifying a growth period during which children are at an increased risk of stunting from lead exposure, our study is able to fill this gap in the literature. In doing so, we demonstrate that nutritional interventions alone are likely not sufficient to reduce later-life stunting in children in developing countries, and that such intervention programs should include reducing early life lead exposure during this critical window of development.

Our finding identifying the heightened susceptibility to the effects of lead on stunting during early childhood is consistent with various studies attempting to provide a mechanistic explanation for the association between early childhood lead exposure and stunting. Animal models suggest the possibility of several mechanistic pathways for this association, including lead-induced reduction in appetite in rats [54], the direct effect of lead on growth plate development [55], and through disruption of early life bone accrual through disruption of osteoclast activity in rats [56].

Studies have consistently shown the possible role of lead alteration of hypothalamic-pituitary axis function through altered release of human growth hormone (GH) and insulin-like growth factor 1 (IGF-1). Rodent studies have found an association between lead and suppressed GH release from the pituitary and decreased IGF-1 concentration [57, 58]. Corroborating these animal models is a recent prospective cohort study that shows an association between increased blood lead level in peripubertal boys and decreased serum IGF-1 [59].

The findings from these animal models and the recent cohort study offer a possible explanation for the inconsistent findings in studies that examine the effect of nutritional interventions on stunting. Previous research has demonstrated that catch-up growth in stunted children is possible both with [60] and without [61, 62] nutritional interventions, yet children who experience early life stunting are consistently shorter than their non-stunted peers. None of these studies adjust for the effect of lead on stunting or HG or IGF-1 secretion, leaving open the possibility that early life lead exposure may play a role in the differential height seen between stunted and non-stunted children even after catch-up growth occurs.

Although our research highlights the importance of concurrent, rather than *in utero*, lead exposure in predicting stunting at age 20 to 40 months, the precise timing of this postnatal lead exposure is unknown. This study makes the assumption that exposure levels during the first 20 to 40 months of age are constant. While the origin of lead exposure in this population is unknown [7], it is likely that child exposure to lead occurs at the household level. Because this research required that all enrolled families remain in the same household for the duration of participation, lead exposure likely remained constant during the study period, thereby minimizing this limitation.

Because nutritional status is a well-documented predictor of early life stunting, our study is limited by our inability to comprehensively adjust for the nutritional status of the child. However, stunting is a form of protein-energy malnutrition [63], and protein deficiency has been shown to be a leading nutritional predictor of stunting [64]. Adjusting for maternal protein intake is therefore likely to capture much of the effect of nutritional status on stunting, with the assumption that children are exposed to similar protein levels as their mothers.

Finally, our study may be limited by unmeasured confounding. While we considered both confounders and predictors of childhood stunting, unmeasured confounding is possible. To account for such unmeasured confounding, we ran a sensitivity analysis adding to our multivariable regression model an indicator for study clinic site. While including this variable adjusted for unmeasured confounders between study sites, it is possible that it resulted in an over-adjusted model. Because inclusion of this study clinic site variable may be either important or superfluous, results are shown for both the clinic adjusted and clinic unadjusted analyses.

This study builds upon existing literature on lead exposure and chronic malnutrition by bridging the gap between environmental health and nutrition. Our use of blood samples to measure child lead exposure allowed us to have the most accurate measure of lead *in utero* and in early childhood [65, 66]. We were additionally able to adjust for a large number of potential confounders of the relationship between lead exposure and stunting as well as predictors of early life stunting. The prospective nature of this study is another strength. Finally, by identifying a critical window of exposure for the association between lead exposure and stunting, this research helps to inform broader public health interventions and provides insight into effective intervention strategies for reducing global stunting rates.

## Conclusions

There is a significant association between stunting and lead exposure at age 20 to 40 months. This association persists, albeit slightly attenuated, when controlling for study clinic site. Additional research is needed to identify when children are at greatest risk of lead-associated stunting during the 20 to 40 month period.

## Additional file

Additional file 1: Table S1. Demographics of total, current and excluded population. **Table S2.** Heavy metal exposure distribution of total, current and excluded population. (DOCX 30 kb)

## Abbreviations

DCH: Dhaka community hospital
DRC-ICP-MS: Dynamic reaction cell-inductively coupled plasma mass spectrometer
EPDS: Edinburgh postnatal depression scale
GH: Human growth hormone
HOME: Home observation for measurement of the environment inventory
ICP-MS: Inductively coupled plasma mass-spectrometry
IGF: Insulin-like growth factor
