# Supplementary material for: Stunting is associated with blood lead concentration among Bangladeshi children aged 2-3 years
Source: Environ Health. 2016 Nov 4;15:103. doi: 10.1186/s12940-016-0190-4 (PMC5096334; doi:10.1186/s12940-016-0190-4)
Supplement: Additional file 1: Table S1. — Demographics of total, current and excluded population. Table S2. Heavy metal exposure distribution of total, current and excluded population. (DOCX 30 kb) [file 12940_2016_190_MOESM1_ESM.docx]

**Additional Materials:**

**Additional Table 1.** Demographics of total, current and excluded population

|  | Total Cohort (n=815) | Current Study Population (n=618) | Excluded Population (n=197) |  | p-value |
| --- | --- | --- | --- | --- | --- |
|  | Number (%) | | |  | Chi-Square Test |
| Clinic |  |  |  |  | *0.06* |
| *Sirajdikhan* | 412 (50.6) | 301 (48.7) | 111 (56.3) |  |  |
| *Pabna* | 403 (49.4) | 317 (51.3) | 86 43.7) |  |  |
| Sex |  |  |  |  |  |
| *Male* | 415 (50.9) | 307 (49.7) | 108 (54.8) |  | *0.21* |
| *Female* | 400 (49.1) | 311 (50.3) | 89 (45.1) |  |  |
| Stunting Status |  |  |  |  |  |
| *Stunted* | 435 (53.4) | 324 (52.4) | 111 (56.3) |  | *0.34* |
| *Not Stunted* | 380 (46.6) | 294 (47.6) | 86 (43.7) |  |  |
| Child Smoke Exposure |  |  |  |  |  |
| *Exposed* | 348 (42.8) | 260 (42.1) | 88 (44.9) |  | *0.49* |
| *Not Exposed* | 466 (57.2) | 358 (57.9) | 108 (55.1) |  |  |
| *Missing* | *1* | *1* | *0* |  |  |
| Mother's education |  |  |  |  |  |
| *Primary or less* | 382 (46.9) | 285 (46.1) | 97 (49.2) |  | *0.45* |
| *Secondary or greater* | 433 (53.1) | 333 (53.9) | 100 (50.8) |  |  |
| Mother's Protein |  |  |  |  | *0.004* |
| *Low* | 206 (25.3) | 139 (22.5) | 67 (34.0) |  |  |
| *Medium* | 423 51.9) | 336 (54.4) | 87 (44.2) |  |  |
| *High* | 186 (22.8) | 143 (23.1) | 43 (21.8) |  |  |
|  | Mean (SD) | | |  | Wilcoxon Rank Sum |
| Birth Gestational Age (weeks) | 38.0 (1.9) | 37.9 (2.0) | 38.17 (1.8) |  | *0.11* |
| *Missing* | *3* | *3* | *0* |  |  |
| Age (months) | 28.1 (2.9) | 28 (2.9) | 28.5 (2.9) |  | *0.03* |
| Mother's Weight at Enrollment (kg) | 46.6 (7.8) | 46.6 (7.6) | 46.6 (8.3) |  | *0.57* |
| Mother's Height (cm) | 151.1 (5.7) | 151.4 (5.6) | 150.4 (5.8) |  | *0.08* |
| Child's Birth Weight (kg) | 2.9 (0.4) | 2.9 (0.4) | 2.9 (0.4) |  | *0.45* |
| *Missing* | *1* | *1* | *0* |  |  |
| Child's Birth Length (cm) | 46.5 (2.6) | 46.6 (2.4) | 46.4 (3.0) |  | *0.45* |
| HOME Score | 42.7 (2.6) | 42.7 (2.6) | 42.7 (2.7) |  | *0.55* |
| *Missing* | *5* | *1* | *4* |  |  |
| Maternal Depression | 15.2 (3.0) | 15.2 (2.8) | 15.1 (3.4) |  | *0.87* |
| *Missing* | *361* | *272* | *89* |  |  |

| **Additional Table 2.** Heavy metal exposure distribution of total, current and excluded population | | | | | |  |
| --- | --- | --- | --- | --- | --- | --- |
|  | Total Cohort (n=815) | Current Study Population (n=618) | Excluded Population (n=197) |  | p-value | |
|  | Median (IQR) | | |  | Wilcoxon Rank Sum | |
| Blood Lead (μg/dL) |  |  |  |  |  | |
| *Umbilical Cord* | 3.1 (1.6-6.5) | 3.1 (1.6-6.3) | 3.63 (1.7-6.7) |  | 0.230 | |
| *Missing* | *7* | - | *7* |  |  | |
| *20-40 Months* | 4.2 (1.7-7.6) | 4.2 (1.7-7.6) | 6.4 (2.4-9.2) |  | 0.550 | |
| *Missing* | *191* | - | *191* |  |  | |
| Average Water Arsenic (μg/L) | 12.5 (1.9-62.9) | 13.43 (1.9-66.3) | 11.7 (1.9-52.3) |  | 0.720 | |
| Average Water Manganese (μg/L) | 625.8 (362.2-1070.0) | 625.5 (368.6-1066.0) | 622.4 (352.8-1070.0) |  | 0.910 | |
